# Analysis of factors associated with IVF/ICSI clinical outcomes in advanced maternal age patients with normal ovarian reserve under the POSEIDON criteria

**DOI:** 10.3389/fendo.2025.1665922

**Published:** 2025-10-13

**Authors:** Yicen Meng, Qianjie Zhang, Yixuan Song, Qian Liu, Wei Li, Jing Yang, Saijiao Li

**Affiliations:** ^1^ Reproductive Medical Center, Renmin Hospital of Wuhan University, Wuhan, China; ^2^ Hubei Clinical Research Center for Assisted Reproductive Technology and Embryonic Development, Wuhan, China; ^3^ The First Clinical College of Wuhan University, Wuhan, China; ^4^ Department of Obstetrics and Gynecology, Renmin Hospital of Wuhan University, Wuhan, China

**Keywords:** advanced maternal age, normal ovarian reserve, POSEIDON criteria, IVF/ICSI, clinical outcome

## Abstract

**Background:**

There is limited research about the pregnancy outcomes of POSEIDON group 2 patients. This study aims to evaluate the factors related to the clinical outcomes of *in vitro* fertilization (IVF) and/or intracytoplasmic sperm injection (ICSI) in POSEIDON group 2 patients, so as to provide clinical guidance to improve clinical outcomes.

**Methods:**

A total of 1,249 IVF/ICSI cycles of POSEIDON group 2 patients were retrospectively analyzed from July 2019 to September 2024. The patients were divided into different groups according to live birth, maternal age, and endometrial thickness (EMT). The clinical outcomes were compared between groups.

**Results:**

Compared with the non-live birth group, maternal age, gonadotropin (Gn) dosage, and Gn total dosage were significantly lower, while EMT was significantly higher in the live birth group (*p* < 0.05). The type of infertility showed statistically significant differences (*p* = 0.013). Multivariate logistic regression analysis indicated that maternal age (OR = 1.195, 95% CI: 1.077–1.327; *p* = 0.001) and EMT on transfer day (OR = 0.887, 95% CI: 0.820–0.959; *p* = 0.003) were independent influencing factors for live birth in POSEIDON group 2 patients. According to the receiver operating characteristic (ROC) curve predictive cutoff value, the patients were divided into group A (age < 38 years, *n* = 249) and group B (age ≥ 38 years, *n* = 155). Group A had a higher human chorionic gonadotropin (hCG)-positive rate (55.8% vs. 34.2%, *p* < 0.001), embryo implantation rate (35.2% vs. 19.2%, *p* < 0.001), clinical pregnancy rate (47.8% vs. 29.7%, *p* < 0.001), and live birth rate (37.3% vs. 20.0%, *p* < 0.001) and a lower early miscarriage rate (12.6% vs. 28.3%, *p* = 0.016) than group B. According to the ROC predictive cutoff value of EMT, group A patients were further divided into group A1 (EMT < 11.65 mm, *n* = 136) and group A2 (EMT ≥ 11.65 mm, *n* = 113). Group B patients were divided into group B1 (EMT < 11.65 mm, *n* = 93) and group B2 (EMT ≥ 11.65 mm, *n* = 62). Compared with group A1 and group A2, group B1 had a lower hCG-positive rate (28.0% vs. 49.3%, *p* = 0.001; 28.0% vs. 63.7%, *p* < 0.001), embryo implantation rate (14.7% vs. 32.2%, *p* < 0.001; 14.7% vs. 40.2%, *p* < 0.001), clinical pregnancy rate (23.7% vs. 41.2%, *p* = 0.006; 23.7% vs. 55.8%, *p* < 0.001), and live birth rate (16.1% vs. 33.8%, *p* = 0.003; 16.1% vs. 41.6%, *p* < 0.001).

**Conclusions:**

Maternal age and EMT on transfer day are independent factors affecting IVF/ICSI clinical outcomes in POSEIDON group 2 patients. Among these, maternal age <38 years or EMT ≥ 11.65 mm indicated better clinical outcomes. Further stratification by age and EMT on transfer day may optimize clinical outcomes in POSEIDON group 2 patients.

## Introduction

With changes in the environment and the delay of female fertility age, the current incidence of infertility has reached as high as 15.5%–25% in China (1). Female fertility is strongly correlated with age. With increasing maternal age and the influence of external environment, the quantity and quality of oocytes of advanced maternal age (AMA) patients deteriorate, resulting in diminished ovarian reserve (DOR) and a progressive decline in female fertility (2). The current demand for assisted reproductive technology (ART) among AMA patients is gradually increasing (3). *In vitro* fertilization (IVF) has become a critical therapeutic option for infertility management. However, previous research have demonstrated that infertility patients aged ≥35 years have lower embryo implantation rates and cumulative live birth rates (CLBR) compared to patients aged <35 years (4). For patients with normal ovarian reserve (NOR) but with advanced maternal age, also named POSEIDON group 2 patients, there is little research about the clinical outcomes of IVF/intracytoplasmic sperm injection (ICSI).

The POSEIDON criteria proposed in 2016 divide patients into groups according to age, anti-Müllerian hormone (AMH), antral follicle count (AFC), and the number of oocytes retrieved in the previous cycle. The introduction of the POSEIDON criteria facilitates the development of more individualized treatment strategies in clinical practice. According to the POSEIDON criteria (5), patients were divided into four groups. POSEIDON group 1 is composed of patients with maternal age <35 years with NOR (AFC ≥ 5, AMH ≥ 1.2 ng/mL), POSEIDON group 2 is made up of patients with maternal age ≥ 35 years with NOR, POSEIDON group 3 consists of patients with maternal age <35 years with DOR (AFC < 5, AMH < 1.2 ng/mL), and POSEIDON group 4 is composed of patients with maternal age ≥ 35 years with DOR. Prognosis was significantly related with age among the four POSEIDON groups (6). Sandro et al. (7) indicated that the incidence rates for each POSEIDON group are as follows: 44.2% (group 1), 36.1% (group 2), 5.2% (group 3), and 14.4% (group 4), which suggested that POSEIDON group 2 accounts for a relatively high proportion within POSEIDON patients. Although POSEIDON group 2 patients have a NOR, some studies have found that the ovarian stimulation response fails to meet expectations. This may be associated with an age-related increased oocyte aneuploidy (8). Sandro et al.’s research confirmed that POSEIDON group 2 patients had a poorer ovarian stimulation response and clinical outcomes compared to POSEIDON group 1 patients, and presented a lower cumulative delivery rate (CDR) and decreased number of oocytes retrieved (9).

Current studies mainly focused on POSEIDON groups 3 and 4, with comparatively limited research on group 2 patients, which exhibit lower pregnancy outcomes (6, 7). Therefore, the objective of our study was to evaluate the factors related to the clinical outcomes of POSEIDON group 2 patients through retrospective analysis, so as to provide clinical guidance to improve clinical outcomes.

## Materials and methods

### Study design and patients

This study retrospectively analyzed 1,249 POSEIDON group 2 patients who underwent IVF/ICSI in the Reproductive Medical Center of Renmin Hospital of Wuhan University from July 2019 to September 2024. The study conformed to the “Declaration of Helsinki for Medical Research Involving Human Subjects”. In addition, this study was approved by the Ethical Committee of Renmin Hospital of Wuhan University (Protocol #: 2023 K-K192). Inclusion criteria for POSEIDON group 2 patients were as follows: (i) age ≥ 35 years; (ii) NOR (AMH ≥ 1.2 ng/mL, AFC ≥ 5) (5, 10); (iii) body mass index (BMI) <28 kg/m^2^; and (iv) fresh embryo transfer cycle, endometrial thickness (EMT) ≥ 7 mm. Patients diagnosed with (i) uterine abnormalities; (ii) endometrial diseases; (iii) endometriosis; (iv) preimplantation genetic testing (PGT), chromosomal abnormalities, or monogenic disorders; (v) an endocrine disorder [diabetes mellitus (DM), hyperprolactinemia, etc.]; and (vi) recurrent pregnancy loss and repeated implantation failure were excluded.

### Clinical setting

In this study, patients received the long protocol, early follicular phase prolonged protocol, or GnRH-ant protocol.

**Long GnRH agonist protocol:** Triptorelin (0.1 mg/day; Decapeptyl, SC, Ferring Pharmaceuticals, Germany) was used in the mid-luteal phase for 10–14 days. Serum hormone levels and ultrasound were performed on the second or the third day of menstrual cycle. If patients reached the downregulation criteria (FSH < 5 U/L, LH < 5 U/L, E_2_ < 50 pg/mL, EMT < 5 mm, and follicle diameter < 5 mm) (11), gonadotropin (Gn) [recombinant follicle-stimulating hormone (r-FSH; Gonal-f^®^, Merck Serono, Germany), recombinant follitropin beta (r-FSH; Puregon^®^, Organon, Oss, Netherlands), human menopausal gonadotropin (hMG; LeBaode^®^, Lizhu Pharmaceutical Factory, China), etc.] was injected at 150–300 IU/day. When more than two follicles’ diameter reached 18 mm or three follicles’ diameter reached 17 mm, final oocyte maturation was triggered by 6,000–10,000 IU of human chorionic gonadotropin (hCG, Lizhu Pharmaceutical Factory, China) or 250 μg of recombinant hCG (r-hCG, Ovidrel^®^, Lizhu Pharmaceutical Factory, China).

**Early follicular phase prolonged protocol:** Gn-releasing hormone agonist [3.75 mg; leuprorelin acetate (Enantone^®^, Lizhu Pharmaceutical Factory, China) or triptorelin acetate (Decapeptyl^®^, Ferring Pharmaceuticals, Switzerland)] was injected intramuscularly on days 1–5 of the menstrual cycle. Ultrasound was performed 28–35 days later. The criteria for downregulation, Gn administration, and trigger protocol were the same as those for the long protocol.

**GnRH-ant protocol:** It is also known as the Gn-releasing hormone antagonist protocol. Gn (150–300 IU/day) was injected on the second or the third day of the menstrual cycle. When the follicle mean diameter reached 14 mm or E_2_ serum levels >300 pg/mL, GnRH-ant (Cetrotide, Merck Serono, Kenilworth, NJ, USA) was injected at 0.25 mg/day until the hCG trigger day. When more than two follicles’ diameter reached 18 mm, final oocyte maturation was triggered by 0.2 mg of GnRH agonist (Decapeptyl 0.2 mg, Ferring International Center SA, Kiel, Germany) and 250 μg of hCG (Lizhu Pharmaceutical Factory, China).

### Oocytes retrieval, fertilization, and embryo transfer

Oocyte retrieval was performed 36–38 h after hCG administration through the guidance of transvaginal ultrasonography. Oocytes were inseminated by either IVF or ICSI according to sperm quality. In this study, all embryo transfers were conducted on days 3–5 after oocyte retrieval and a maximum of two embryos were transferred. A high-quality embryo on day 3 was defined as an embryo with seven or eight blastomeres, no multinucleation, and <20% fragmentation. The blastocyst was graded according to the Gardner scoring standard. Those achieving ≥3BB on day 5 or ≥4BB on day 6 were defined as high-quality blastocysts (12). Fresh embryo transfer was cancelled under the following circumstances (13): (i) E_2_ > 18,350 pmol/L on hCG day in the GnRH-ant protocol; (ii) high risk of ovarian hyperstimulation syndrome (OHSS); and (iii) progesterone >5.57 nmol/L on hCG day.

### Clinical outcomes and follow-up

The luteal support was started on the day of oocyte retrieval. Intramuscular progesterone (20 mg once daily), vaginal progesterone sustained-release gel (Crinone 8%, 90 mg once daily), and oral progesterone (Dydrogesterone, 20 mg twice daily) were administered for luteal support. A serum β-hCG test was performed 12 days after embryo transfer. Ultrasound was performed 30 days after transplantation. The luteal phase support was administered until 9–12 weeks of gestation. Serum β-hCG > 10 IU/L was considered a chemical pregnancy. Clinical pregnancy was defined as the presence of a gestational sac with a fetal heart under ultrasonography 30 days after embryo transfer. Early miscarriage was defined as embryo loss before 12 weeks of pregnancy (14).

### Statistical analysis

SPSS 26.0 (IBM Corp., USA) was used for data analysis. A receiver operating characteristic (ROC) curve was used to analyze the predictive value of factors on live birth. The predictive cutoff value was determined according to the maximum value of the Youden index, and the Youden index is equal to sensitivity + specificity − 1. Measurement data conforming to a normal distribution were expressed as mean ± SD. The independent-sample *t*-test and one-way analysis of variance (ANOVA) were used to compare variables between groups. Categorical variables were compared using the chi-square test or Fisher’s precision probability test. The multivariate logical regression model is used to analyze influencing factors of clinical pregnancy. Bonferroni correction was used for multiple comparisons of data. *p*-value < 0.05 was considered statistically significant.

## Results

**Figure 1:**
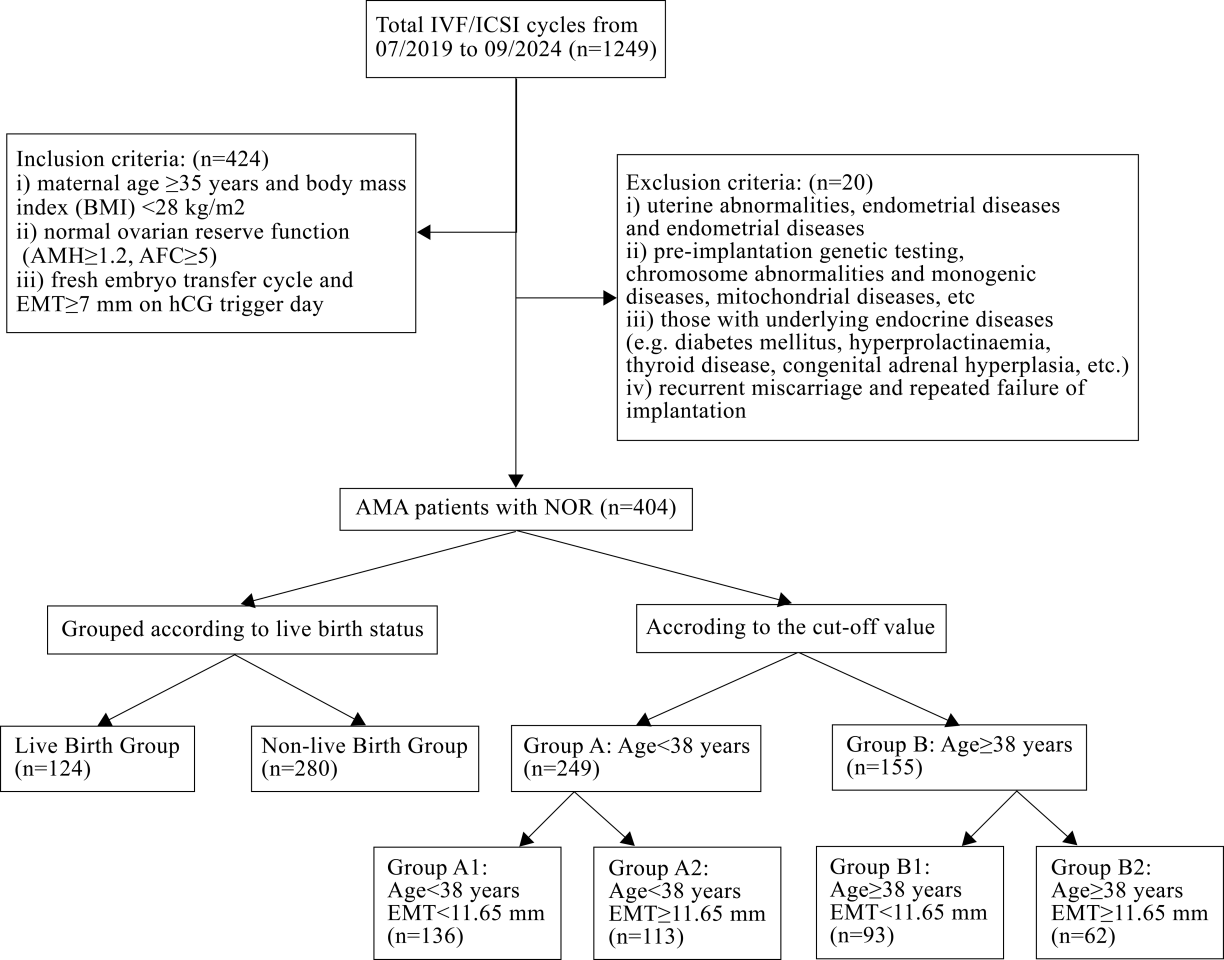
The flowchart of the study.

A total of 404 POSEIDON group 2 patients were included in this study according to the above inclusion and exclusion criteria, as shown in **Figure 1**.

According to clinical outcomes, patients were divided into 2 groups: the live birth group (*n* = 124) and the non-live birth group (*n* = 280). The comparison of baseline characteristics between the two groups is shown in **Table 1**. The results indicated that compared to the non-live birth group, age, Gn dosage, and Gn total dosage in the live birth group were significantly lower (*p* < 0.05), while the EMT on transfer day was significantly higher (*p* < 0.05). The type of infertility showed statistically significant differences (*p* = 0.013). There was no significant difference in other characteristics between the two groups.

**Table 1:** The comparison of baseline characteristics and ovulation induction between the live birth group and the non-live birth group.

Variables	Live birth group (*n* = 124)	Non-live birth group (*n* = 280)	*p*-value
Age (years)	36.74 ± 1.81	37.75 ± 2.63	<0.001
BMI (kg/m^2^)	22.57 ± 2.21	22.17 ± 2.42	0.104
Duration of infertility (years)	3.60 ± 3.46	3.76 ± 3.02	0.662
Type of infertility			
Primary infertility	45/124 (36.3)	68/280 (24.3)	0.013
Secondary infertility	79/124 (63.7)	212/280 (75.7)	
Basal FSH (mIU/L)	7.87 ± 2.08	8.29 ± 3.88	0.165
Basal LH (mIU/L)	3.60 ± 1.88	3.91 ± 1.97	0.134
Basal E_2_ (pg/mL)	43.43 ± 15.06	43.76 ± 16.85	0.834
AMH (ng/mL)	2.47 ± 0.95	2.37 ± 0.91	0.311
AFC	13.44 ± 4.42	13.05 ± 4.66	0.431
Factors of infertility			
Tubal factors	86/124 (69.4)	183/280 (65.3)	0.513
Male factors	18/124 (14.5)	38/280 (13.6)	
Unknown factors	20/124 (16.1)	59/280 (21.1)	
Controlled ovarian stimulation (COS) protocol			
GnRH-ant protocol	47/124 (37.9)	130/280 (46.4)	0.162
Long protocol	43/124 (34.7)	73/280 (26.1)	
Early follicular phase prolonged protocol	34/124 (27.4)	77/280 (27.5)	
Type of fertilization			
IVF	99/124 (79.8)	227/280 (81.1)	0.728
ICSI	19/124 (15.3)	44/280 (15.7)	
IVF + Rescue ICSI	6/124 (4.8)	9/280 (3.2)	
Gn dosage (IU)	214.48 ± 51.84	227.58 ± 53.83	0.021
Gn total dosage (IU)	2,289.96 ± 634.96	2,440.98 ± 750.35	0.038
Gn duration (days)	10.19 ± 2.09	10.36 ± 2.43	0.452
EMT (mm)	11.97 ± 2.90	11.09 ± 2.29	0.003
Estradiol level on hCG day (pg/mL)	2,222.66 ± 824.30	2,298.46 ± 910.48	0.410
Progesterone level on hCG day (ng/mL)	0.74 ± 0.23	0.77 ± 0.24	0.345
LH level on hCG day (mIU/L)	1.74 ± 1.38	1.90 ± 1.71	0.348

Datae are shown as mean ± SD or *n* (%).

The comparison of laboratory parameters between the live birth group and the non-live birth group is shown in **Table 2**. There was no significant difference in the laboratory parameters between the two groups.

**Table 2:** The comparison of laboratory parameters between the live birth group and the non-live birth group.

Variables	Live birth group (*n* = 124)	Non-live birth group (*n* = 280)	*p*-value
Number of oocytes retrieved	9.65 ± 4.44	9.42 ± 4.66	0.640
2PN (two pronuclei) fertility rate	61.55 ± 22.55	61.20 ± 22.97	0.887
2PN cleavage rate	93.78 ± 22.11	89.49 ± 28.13	0.100
Number of high-quality embryos	3.44 ± 2.44	3.33 ± 2.69	0.685
High-quality embryo rate	49.35 ± 27.10	48.06 ± 29.19	0.666
Number of embryo transfer	1.62 ± 0.49	1.61 ± 0.50	0.847
Types of embryo transfer			
Cleavage-stage embryo	95/124 (76.6)	204/280 (72.9)	0.427
Blastocyst-stage embryo	29/124 (23.4)	76/280 (27.1)	

Data are shown as mean ± SD or *n* (%).

Multivariate logistic regression was used to analyze the effects of maternal age, EMT on transfer day, Gn dosage, Gn total dosage, and type of infertility on live birth. As shown in **Table 3**, logistic regression analysis showed that age (*p* = 0.001), EMT (*p* = 0.003), and type of infertility (*p* = 0.004) on transfer day were independent related factors of live birth in POSEIDON group 2 patients. The Hosmer–Lemeshow test (H–L test) was used to verify the calibration accuracy of the model. The H–L test revealed *p*-values of 0.135, 0.327, and N/A for maternal age, EMT, and type of infertility, respectively (*p* > 0.05). Combined with the results of the multivariate logistic regression analysis, it indicated that age and EMT had good clinical discrimination and calibration. However, as the type of infertility is a categorical variable, the sample distribution resulted in low expected frequencies in the H–L test, preventing its valid computation.

**Table 3:** Multivariate logistic regression analysis of live birth in POSEIDON group 2 patients.

Variables	*B*	SE	Wald χ^2^	Df	*p*-value	Adjusted OR	95% CI
Age (years)	0.178	0.053	11.219	1	0.001	1.195	1.077–1.327
Gn dosage (IU)	0.001	0.002	0.180	1	0.671	1.001	0.997–1.005
Gn total dosage (IU)	0.000	0.000	2.241	1	0.134	1.000	1.000–1.001
EMT (mm)	−0.120	0.040	8.958	1	0.003	0.887	0.820–0.959
Type of infertility	−0.611	0.210	8.445	1	0.004	0.543	0.359–0.820

**Figure 2:**
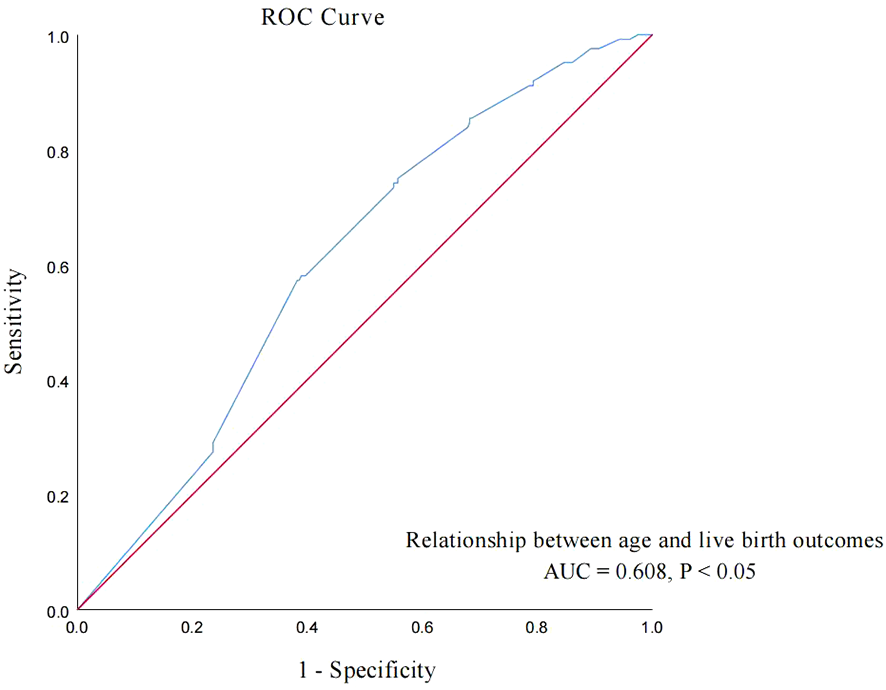
ROC curve: analyze the relationship between age and live birth of POSEIDON group 2 patients.

To further explore the relationship between age and live birth of POSEIDON group 2 patients, an ROC curve was used and the results are shown in **Figure 2**. The area under the curve (AUC) of the age for predicting the live birth was 0.608 (95% CI: 0.558–0.658, *p* < 0.001). According to the Youden index, the cutoff value of the ROC curve is 37.915, and age has the highest sensitivity and specificity (75.0% and 44.3%, respectively) in predicting live birth. According to the cutoff value, patients were divided into group A (age < 38 years, *n* = 249) and group B (age ≥ 38 years, *n* = 155).

The comparison of baseline characteristics and COS characteristics of patients between the two groups is shown in **Table 4**. Compared with group B, BMI, Gn dosage, and LH level on hCG day in group A significantly decreased (*p* < 0.05), and AMH, AFC, and Gn duration significantly increased (*p* < 0.05). The type of infertility (*p* = 0.005), factors of infertility (*p* = 0.026), and COS protocol (*p* = 0.005) showed statistically significant differences. There was no significant difference in other characteristics between groups A and B.

**Table 4:** The comparison of baseline characteristics and ovulation induction between group A and group B.

Variables	Group A Age < 38 years (*n* = 249)	Group B Age ≥ 38 years (*n* = 155)	*p*-value
BMI (kg/m^2^)	22.07 ± 2.49	22.50 ± 2.20	0.020
Infertility duration (years)	3.57 ± 2.84	3.97 ± 3.43	0.098
Type of infertility			
Primary infertility	82/249 (32.9)	31/155 (20.0)	0.005
Secondary infertility	167/249 (67.1)	124/155 (80.0)	
Basal FSH (mIU/L)	8.09 ± 2.46	8.39 ± 4.84	0.283
Basal LH (mIU/L)	3.91 ± 2.07	3.77 ± 1.76	0.374
Basal E_2_ (pg/mL)	42.92 ± 15.84	44.89 ± 17.41	0.126
AMH (ng/mL)	2.48 ± 0.92	2.26 ± 0.90	0.002
AFC	13.87 ± 4.84	12.03 ± 4.03	<0.001
Factors of infertility			
Tubal factors	163/249 (65.4)	106/155 (68.4)	0.026
Male factors	43/249 (17.3)	13/155 (8.4)	
Unknown factors	43/249 (17.3)	36/155 (23.2)	
COS protocol			
GnRH-ant protocol	96/249 (38.6)	81/155 (52.3)	0.005
Long protocol	85/249 (34.1)	31/155 (20.0)	
Early follicular phase prolonged protocol	68/249 (27.3)	43/155 (27.7)	
Type of fertilization			
IVF	197/249 (79.1)	129/155 (83.2)	0.500
ICSI	41/249 (16.5)	22/155 (14.2)	
IVF + Rescue ICSI	11/249 (4.4)	4/155 (2.6)	
Gn dosage (IU)	212.71 ± 50.63	243.35 ± 52.76	<0.001
Gn total dosage (IU)	2,390.36 ± 763.13	2,447.34 ± 684.52	0.308
Gn duration (days)	10.55 ± 2.26	10.02 ± 2.49	0.005
EMT (mm)	11.39 ± 2.35	11.04 ± 2.54	0.070
Estradiol level on hCG day (pg/mL)	2,337.03 ± 849.79	2,208.79 ± 952.58	0.065
Progesterone level on hCG day (ng/mL)	0.78 ± 0.23	0.75 ± 0.24	0.108
LH level on hCG day (mIU/L)	1.75 ± 1.65	2.04 ± 1.65	0.023

Data are shown as mean ± SD or *n* (%).

The comparison of laboratory parameters and clinical outcomes between group A and group B is shown in **Table 5**. The high-quality embryo rate and number of embryo transfer in group A were significantly lower than those in group B (*p* < 0.05). Compared to group B, the number of oocytes retrieved (*p* < 0.05), 2PN cleavage rate (*p* < 0.05), hCG-positive rate (*p* < 0.001), embryo implantation rate (*p* < 0.001), clinical pregnancy rate (*p* < 0.001), and live birth rate (*p* < 0.001) in group A were significantly higher, while the early miscarriage rate (*p* = 0.016) was significantly lower. There was no significant difference in other characteristics between group A and group B.

**Table 5:** The comparison of laboratory parameters and clinical outcomes between group A and group B.

Variables	Group A Age < 38 years (*n* = 249)	Group B Age ≥ 38 years (*n* = 155)	*p*-value
Number of oocytes retrieved	9.79 ± 4.98	8.99 ± 3.99	0.026
2PN fertility rate	60.72 ± 22.73	62.06 ± 23.08	0.455
2PN cleavage rate	92.74 ± 23.27	86.67 ± 31.67	0.004
Number of high-quality embryos	3.30 ± 2.70	3.41 ± 2.56	0.598
High-quality embryo rate	45.52 ± 28.63	52.32 ± 28.60	0.002
Number of embryo transfer	1.57 ± 0.51	1.68 ± 0.48	0.003
Types of embryo transfer			
Cleavage-stage embryo	183/249 (73.5)	116/155 (74.8)	0.764
Blastocyst-stage embryo	66/249 (26.5)	39/155 (25.2)	
hCG-positive rate	139/249 (55.8)	53/155 (34.2)	<0.001
Implantation rate	138/392 (35.2)	50/260 (19.2)	<0.001
Clinical pregnancy rate	119/249 (47.8)	46/155 (29.7)	<0.001
Early miscarriage rate	15/119 (12.6)	13/46 (28.3)	0.016
Live birth rate	93/249 (37.3)	31/155 (20.0)	<0.001

Data are shown as mean ± SD or *n* (%).

hCG-positive rate = number of hCG-positive cycles/number of transfer cycles × 100%.

Implantation rate = number of pregnancy sacs/number of transferred embryos × 100%.

Clinical pregnancy rate = number of clinical pregnancy cycles/number of transfer cycles × 100%.

Early miscarriage rate = number of early miscarriage cycles/clinical pregnancy cycles × 100%.

Live birth rate = number of live birth cycles/number of transfer cycles × 100%.

**Figure 3:**
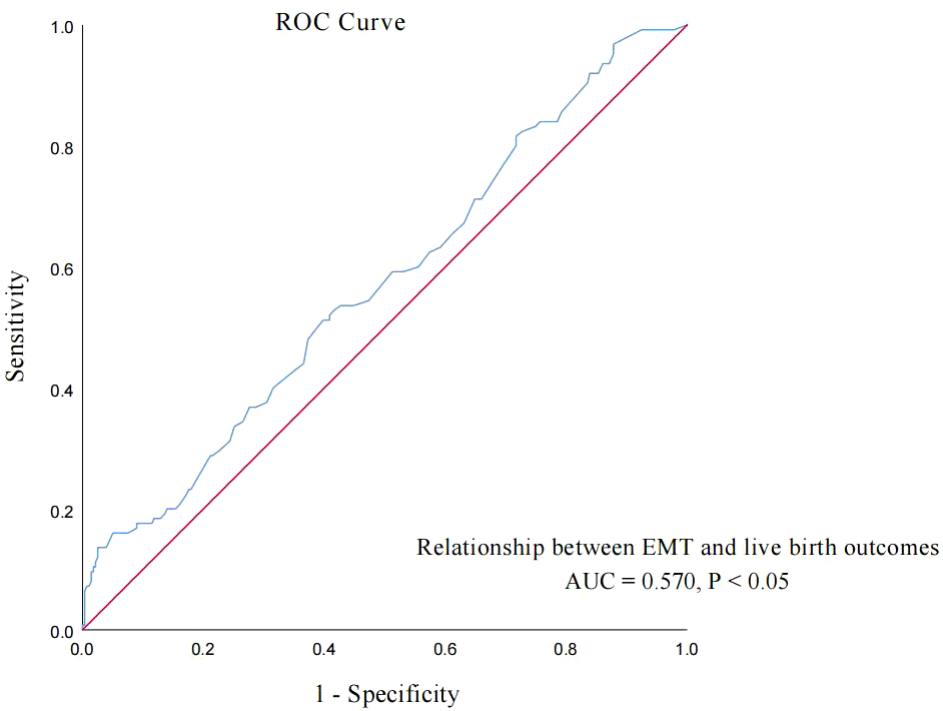
ROC curve: analyze the relationship between EMT on transfer day and live birth of POSEIDON group 2 patients.

EMT on transfer day was also an independent influencing factor of live birth in POSEIDON group 2 patients. Then, the ROC curve was used to analyze the relationship between the EMT on transfer day and live birth of POSEIDON group 2 patients, and the results are shown in the curve in **Figure 3**. The AUC of the EMT on transfer day for predicting the live birth was 0.570 (95% CI: 0.515–0.626, *p* = 0.014). According to the Youden index, the cutoff value of the ROC curve is 11.65 mm, and the EMT has the highest sensitivity and specificity (51.2% and 60.2%, respectively) in predicting live birth. According to the cutoff value, group A patients were further divided into group A1 (EMT < 11.65 mm, *n* = 136) and group A2 (EMT ≥ 11.65 mm, *n* = 113). Group B patients were divided into group B1 (EMT < 11.65 mm, *n* = 93) and group B2 (EMT ≥ 11.65 mm, *n* = 62).

A comparison of four groups was carried out to examine baseline characteristics and COS characteristics, which are shown in **Table 6**. The type of infertility (*p* = 0.002) showed statistically significant differences in group A2 and group B1. The AMH in group A2 was higher than that in group B1 (*p* < 0.001), while the LH level on hCG day was lower than that in group B1 (*p* = 0.002). The AFC in group B1 and group B2 was lower than that in group A1 and group A2 (*p* < 0.008). There was no significant difference in other characteristics between the subgroups at the same age.

**Table 6:** The comparison of baseline characteristics between group A1, group A2, group B1, and group B2.

Variables	Group A1 Age < 38 years EMT < 11.65 mm (*n* = 136)	Group A2 Age < 38 years EMT ≥ 11.65 mm (*n* = 113)	Group B1 Age ≥ 38 years EMT < 11.65 mm (*n* = 93)	Group B2 Age ≥ 38 years EMT ≥ 11.65 mm (*n* = 62)
BMI (kg/m^2^)	22.13 ± 2.55	22.21 ± 2.31	22.38 ± 2.34	22.70 ± 1.99
Duration of infertility (years)	3.33 ± 2.81	3.85 ± 3.11	3.92 ± 3.60	3.98 ± 3.26
Type of infertility				
Primary infertility	37/136 (27.2)	45/113 (39.8)	18/93 (19.4)^b^	13/62 (21.0)
Secondary infertility	99/136 (72.8)	68/113 (60.2)	75/93 (80.6)^b^	49/62 (79.0)
Basal FSH (mIU/L)	8.08 ± 2.20	8.06 ± 2.62	8.64 ± 5.65	7.81 ± 2.34
Basal LH (mIU/L)	3.96 ± 2.15	3.71 ± 1.95	3.67 ± 1.61	3.91 ± 1.92
Basal E_2_ (pg/mL)	43.77 ± 16.39	41.92 ± 14.27	45.05 ± 18.27	44.62 ± 16.31
AMH (ng/mL)	2.45 ± 0.95	2.54 ± 0.90	2.22 ± 0.88^b^	2.33 ± 0.91
AFC	13.68 ± 4.99	14.03 ± 4.57	12.13 ± 4.23^ab^	12.05 ± 3.62^ab^
Type of fertilization				
IVF	109/136 (80.1)	88/113 (77.9)	75/93 (80.7)	54/62 (87.1)
ICSI	22/136 (16.2)	19/113 (16.8)	15/93 (16.1)	7/62 (11.3)
IVF + Rescue ICSI	5/136 (3.7)	6/113 (5.3)	3/93 (3.2)	1/62 (1.6)
Estradiol level on hCG day (pg/mL)	2,305.13 ± 838.31	2,349.10 ± 859.51	2,246.60 ± 937.53	2,117.72 ± 931.37
Progesterone level on hCG day (ng/mL)	0.77 ± 0.24	0.78 ± 0.23	0.75 ± 0.23	0.72 ± 0.25
LH level on hCG day (mIU/L)	1.88 ± 1.78	1.59 ± 1.38	2.11 ± 1.56^b^	1.84 ± 1.67

Data are shown as mean ± SD or *n* (%).

After Bonferroni correction, ^a^ *p* < 0.008 compared with group A1, ^b^ *p* < 0.008 compared with group A2.

The comparison of laboratory parameters and clinical outcomes between 4 groups is shown in **Table 7**. The results indicated that the high-quality embryo rate in group B1 was significantly higher than that in group A2 (*p* = 0.002). The hCG-positive rate (*p* = 0.001; *p* < 0.001), embryo implantation rate (*p* < 0.001; *p* < 0.001), clinical pregnancy rate (*p* = 0.006; *p* < 0.001), and live birth rate (*p* = 0.003; *p* < 0.001) were significantly lower than those in group A1 and group A2. There was no significant difference in early miscarriage rate and other characteristics between the subgroups either.

**Table 7:** The comparison of laboratory parameters and clinical outcomes between group A1, group A2, group B1, and group B2.

Variables	Group A1 Age < 38 years EMT < 11.65 mm (*n* = 136)	Group A2 Age < 38 years EMT ≥ 11.65 mm (*n* = 113)	Group B1 Age ≥ 38 years EMT < 11.65 mm (*n* = 93)	Group B2 Age ≥ 38 years EMT ≥ 11.65 mm (*n* = 62)
Number of oocytes retrieved	9.81 ± 4.79	9.73 ± 5.10	9.04 ± 3.88	9.03 ± 4.06
2PN fertility rate	60.04 ± 22.44	61.45 ± 23.28	62.46 ± 22.80	62.11 ± 22.86
2PN Cleavage rate	93.49 ± 21.68	92.25 ± 24.68	86.71 ± 31.41	88.42 ± 30.12
Number of high-quality embryos	3.32 ± 2.58	3.35 ± 2.79	3.56 ± 2.57	3.16 ± 2.42
High-quality embryo rate	46.89 ± 29.17	45.75 ± 27.47	54.78 ± 28.11^b^	47.34 ± 28.56
Number of embryo transfer	1.57 ± 0.51	1.58 ± 0.49	1.68 ± 0.47	1.68 ± 0.41
Types of embryo transfer				
Cleavage-stage embryo	99/136 (72.8)	84/113 (74.3)	70/93 (75.3)	46/62 (74.2)
Blastocyst-stage embryo	37/136 (27.2)	29/113 (25.7)	23/93 (24.7)	16/62 (25.8)
hCG-positive rate	67/136 (49.3)	72/113 (63.7)	26/93 (28.0)^ab^	27/62 (43.5)
Implantation rate	66/205 (32.2)	72/179 (40.2)	23/156 (14.7)^ab^	27/104 (26.0)
Clinical pregnancy rate	56/136 (41.2)	63/113 (55.8)	22/93 (23.7)^ab^	24/62 (38.7)
Early miscarriage rate	7/56 (12.5)	8/63 (12.7)	7/22 (31.8)	6/24 (25.0)
Live birth rate	46/136 (33.8)	47/113 (41.6)	15/93 (16.1)^ab^	16/62 (25.8)

Data are shown as mean ± SD or *n* (%).

After Bonferroni correction, ^a^ *p* < 0.008 compared with group A1, ^b^ *p* < 0.008 compared with group A2.

## Discussion

In this study, we investigated the factors related to the clinical outcomes of IVF and/or ICSI in POSEIDON group 2 patients and found that the age and EMT on transfer day were independent related factors of live birth in POSEIDON group 2 patients. Our study demonstrated that despite more embryos transferred number in the age ≥ 38 years group, the age < 38 years group showed significantly higher hCG-positive rate, embryo implantation rate, clinical pregnancy rate, and live birth rate. Furthermore, the hCG-positive rate, embryo implantation rate, clinical pregnancy rate, and live birth rate in POSEIDON group 2 patients aged ≥38 with EMT < 11.65 mm were significantly lower than those patients aged <38 with EMT ≥ 11.65 mm. This was the first study to conduct stratification and subgroup analysis of age and EMT on transfer day in POSEIDON group 2 patients using the cutoff value of ROC curves. Our results demonstrate the important predictive value of maternal age and EMT on transfer day for clinical outcomes in POSEIDON group 2 patients, and POSEIDON group 2 patients with EMT ≥ 11.65 mm on transfer day who underwent IVF before the age of 38 will achieve better clinical outcomes.

Maternal age is one of the most critical factors in predicting IVF pregnancy outcomes. Our study demonstrated that age was an independent related factor of clinical pregnancy in POSEIDON group 2 patients. Similar to the results of our study, several studies demonstrated that POSEIDON group 2 patients had a low IVF clinical pregnancy rate and a high miscarriage rate compared to patients aged <35 (15, 16). The incidence rate of adverse pregnancy outcomes increased significantly with maternal age, especially in those aged ≥40 (17–19). Meta-analyses indicated that AMA leads to a decrease in fertility and increases the risk of early pregnancy loss by elevating the incidence of chromosomal abnormalities and genetic disorders (20). Furthermore, studies demonstrated that the pregnancy rate and CLBR were significantly higher in young POSEIDON (group 1 and 3) groups than in the age ≥35 years POSEIDON (group 2 and 4) groups, and repeated ovarian stimulation could compensate for reduced oocyte quantity and quality in young POSEIDON groups (21, 22). Reig et al. (23) divided patients undergoing PGT into five groups by patient age, and statistical analysis showed that the embryo implantation rate was negatively correlated with age. However, there was no significant difference in clinical pregnancy rate among different age groups. It was different from our results. This may be due to research patients’ different inclusion criteria and the limited sample size in the AMA group.

The ovarian reserve declines rapidly in patients with maternal age ≥38. This was related to the increasing oocyte aneuploidy rate (15). Some studies indicated that there was an increased incidence of chromosomal abnormalities and chromosomal aneuploidy with the advancing maternal age. In patients with maternal age ≥38, the chromosomal abnormality rate exceeded 60% (24, 25). Huang et al. (26) demonstrated that oocytes are more prone to meiotic chromosome segregation errors as female patients age. This leads to aneuploidy and contributes to decreased fertility and adverse reproductive outcomes. This may be an important factor contributing to the high early miscarriage rate and low embryo implantation rate in patients aged ≥38 years. Consistent with the previous study, in our study, the age of POSEIDON group 2 patients was further stratified by ROC curve and 38 years old was used as a cutoff point. Compared with patients aged <38 years, those aged ≥38 years had a lower embryo implantation rate and clinical pregnancy rate, but a higher early miscarriage rate. This may be related to the significant increase in embryonic aneuploidy and the decline in embryo quality in those with maternal age ≥38. In clinical practice, particular attention should be paid to patients aged ≥38 in POSEIDON group 2, and preimplantation genetic testing for aneuploid (PGT-A) or other management methods should be considered to optimize clinical outcomes.

EMT is a key monitored indicator in the IVF/ICSI process and an indirect indicator of endometrial receptivity (27). Current research predominantly focus on POSEIDON groups 1 and 3, while there is no targeted analysis on the relationship between EMT and clinical outcomes in group 2 patients. A linear regression study demonstrated that patients with EMT ≥ 11 mm on transfer day exhibited a significantly higher clinical pregnancy rate and a lower ectopic pregnancy rate than patients with EMT < 11 mm on transfer day (27). Some studies have also demonstrated that thin endometrium adversely affects embryo implantation and leads to poor clinical outcomes. It was presented with a lower pregnancy rate, implantation rate, and live birth rate, as well as an increased risk of adverse neonatal clinical outcomes (28–30). A study also found that EMT ≤ 7 mm can affect endometrial receptivity, which leads to lower embryo implantation rates and clinical pregnancy rates for assisted reproductive treatment (31). Mahutte et al. (32) found that the live birth rates increased significantly with EMT up to 10–12 mm in fresh embryo transfers. However, after these thresholds, live birth rates plateaued. Consistent with the previous study, the EMT on transfer day of POSEIDON group 2 patients was further stratified by ROC curve in our study and an EMT of 11.65 mm was used as a cutoff point. Interestingly, our study demonstrates that a thicker EMT on transfer day may partially ameliorate the negative impact of AMA on clinical outcomes, as evidenced by an increase in the hCG-positive rate, embryo implantation rate, clinical pregnancy rate, and live birth rate. However, these currently lack statistical significance, which may be related to the limited sample size. These findings suggest that the EMT > 11.65 mm on transfer day may serve as a decisive cutoff for determining the suitability of fresh embryo transfer in clinical practice for POSEIDON group 2 patients, which may help to improve the clinical outcomes of these patients.

### Limitation

The main limitation of our study is that this is a retrospective study with inevitable selective bias. Although the cutoff value of the ROC curve and most clinical outcomes in this study showed statistically significant differences, the relatively small sample size may limit the stability of the results. Furthermore, the ROC cutoff value provides a useful predictive benchmark within our study; it remains an exploratory finding. Large-sample prospective trials and multicenter randomized controlled trials are still needed for further verification and clarification of the specific mechanisms in the future.

## Conclusion

In conclusion, the results demonstrate the important predictive value of maternal age and EMT on transfer day for clinical outcomes in POSEIDON group 2 patients. Age ≥38 and EMT < 11.65 mm on transfer day in POSEIDON group 2 patients should be considered as critical nodes that affect clinical outcomes and should be paid specific attention. Meanwhile, we may also moderately improve clinical outcomes in POSEIDON group 2 patients aged ≥38 years by increasing EMT > 11.65 mm on transfer day. This may provide clinical guidance to improve clinical outcomes for POSEIDON group 2 patients undergoing IVF/ICSI treatment, and it will be a focus of our future research.

## Data Availability

The raw data supporting the conclusions of this article will be made available by the authors, without undue reservation.
